# Glibenclamide inhibits cell growth by inducing G0/G1 arrest in the human breast cancer cell line MDA-MB-231

**DOI:** 10.1186/2050-6511-14-6

**Published:** 2013-01-11

**Authors:** Mariel Núñez, Vanina Medina, Graciela Cricco, Máximo Croci, Claudia Cocca, Elena Rivera, Rosa Bergoc, Gabriela Martín

**Affiliations:** 1 Radioisotopes Laboratory, School of Pharmacy and Biochemistry, University of Buenos Aires, Buenos Aires, Argentina; 2 Institute of Immunooncology Dr. EJV Crescenti, Buenos Aires, Argentina; 3 Institute of Health Sciences Barceló, Buenos Aires, Argentina

**Keywords:** Glibenclamide, Potassium channels, MDA-MB-231, Cytostatic effect

## Abstract

**Background:**

Glibenclamide (Gli) binds to the sulphonylurea receptor (SUR) that is a regulatory subunit of ATP-sensitive potassium channels (K_ATP_ channels). Binding of Gli to SUR produces the closure of K_ATP_ channels and the inhibition of their activity. This drug is widely used for treatment of type 2-diabetes and it has been signaled as antiproliferative in several tumor cell lines. In previous experiments we demonstrated the antitumoral effect of Gli in mammary tumors induced in rats. The aim of the present work was to investigate the effect of Gli on MDA-MB-231 breast cancer cell proliferation and to examine the possible pathways involved in this action.

**Results:**

The mRNA expression of the different subunits that compose the K_ATP_ channels was evaluated in MDA-MB-231 cells by reverse transcriptase-polymerase chain reaction. Results showed the expression of mRNA for both pore-forming isoforms Kir6.1 and Kir6.2 and for the regulatory isoform SUR2B in this cell line. Gli inhibited cell proliferation assessed by a clonogenic method in a dose dependent manner, with an increment in the population doubling time. The K_ATP_ channel opener minoxidil increased clonogenic proliferation, effect that was counteracted by Gli. When cell cycle analysis was performed by flow cytometry, Gli induced a significant cell-cycle arrest in G0/G1 phase, together with an up-regulation of p27 levels and a diminution in cyclin E expression, both evaluated by immunoblot. However, neither differentiation evaluated by neutral lipid accumulation nor apoptosis assessed by different methodologies were detected. The cytostatic, non toxic effect on cell proliferation was confirmed by removal of the drug.

Combination treatment of Gli with tamoxifen or doxorubicin showed an increment in the antiproliferative effect only for doxorubicin.

**Conclusions:**

Our data clearly demonstrated a cytostatic effect of Gli in MDA-MB-231 cells that may be mediated through K_ATP_ channels, associated to the inhibition of the G1-S phase progression. In addition, an interesting observation about the effect of the combination of Gli with doxorubicin leads to future research for a potential novel role for Gli as an adjuvant in breast cancer treatment

## Background

Sulphonylureas are used to increase insulin secretion in patient with type 2-diabetes due to their direct action on pancreatic β cells. These drugs bind to the β cell sulphonylurea receptor (SUR) that is a regulatory subunit of ATP-sensitive potassium channels (K_ATP_ channels) [1-3]. K_ATP_ channels regulate the transport of potassium ions through cell membranes. A diverse group of compounds can bind to K_ATP_ channels causing them to open or close. The opening of potassium channels in the cell membrane produces a hyperpolarization of membrane potential. These channels are hetero-octameric complexes that consist of two rings: an inner ring of four inwardly rectifying K^+^ channels (Kir6.X) that forms the pore through which potassium ions pass, and an outer ring that comprises four SUR subunits. Two isoforms have been described for Kir6.X (Kir6.1 and Kir6.2) and also for SUR (SUR1 and SUR2; SUR2 also has two splice variants, SUR2A and SUR2B) [4]. In pancreatic β cells binding of sulphonylureas to SURs produce the closure of K_ATP_ channels reducing cellular potassium efflux thus favoring membrane depolarization, the induction of Ca^2+^ influx, and insulin secretion [1-3].

Glibenclamide (Gli), a diarylsulphonilurea that blocks specifically K_ATP_ channels, is widely employed in the treatment of diabetic patients [5], but various reports also described its antiproliferative effect in different neoplastic cell lines [6,7]. Additionally, the inhibition of other classes of potassium channels also leads to a decrease of proliferation in normal and cancer cells [8,9].

Breast cancer is the neoplastic disease most frequently observed in women all over the world [10-12]. A high proportion of mammary tumors are positive for estrogen receptors α (ERα) and consequently antihormonal therapy is indicated. The selective estrogen receptor modulator tamoxifen continues to be the drug regularly used in patients harboring this kind of tumors due to its efficacy and low toxicity [13]. However, approximately 30% of ERα (+) tumors do not respond to tamoxifen or develop resistance in the course of the treatment. In addition, it is known that approximately 30% of tumors do not express ERα [14]. Although a large number of drugs have been developed for the treatment of ERα (−) tumors, most of them give rise to important toxic effects. In order to attain better therapeutic effectiveness, combination cytotoxic treatments for aggressive cancers have been employed in clinics. The simultaneous use of drugs with different molecular targets can delay the emergence of chemoresistance whereas when drugs are directed to the same cellular pathway they could work synergically for higher efficacy and selectivity. However, combination therapy may also increase toxicity [15]. Doxorubicin is considered a highly effective agent in the treatment of aggressive breast cancer patients sometimes combined with cyclophosphamide, taxanes and/or 5-fluorouracil; however, resistance to doxorubicin is common [16,17]. The search for effective drugs with low side effects is still a challenge to researchers.

MDA-MB-231 cell line derived from a human breast carcinoma that do not express ERα, is often used as an experimental non hormone-dependent tumor model [18,19]. The objective of the present work was to investigate the effect of Gli on MDA-MB-231 cells proliferation and to examine the possible pathways involved in this action.

### Material and methods

#### Materials

MBA-MB-231 cells were obtained from American Type Culture Collection (ATCC). Gli was kindly provided by Investi-Farma SA, Buenos Aires, Argentina. Tamoxifen (Tam) was a gift from Gador Laboratories SA, Buenos Aires, Argentina. RPMI 1640 medium and fetal bovine serum (FBS) were purchased from GIBCO, Invitrogen, CA, USA. Ribonuclease, propidium iodine, 3,3^′^dihexyloxacarbocyanine iodide (DiOC6), saponine, FITC-labeled anti-rabbit, 5-bromo-2^′^-deoxyuridine (BrdU), mouse anti-BrdU monoclonal antibody, FITC-conjugated anti-mouse IgG, 4^′^,6-Diamidino-2-phenyindole, dilactate (DAPI), 5-bromo-4-chloro-3-indolyl-β-d-galactoside (X-gal), mouse anti-β-actin polyclonal antibody, p27^Kip1^ monoclonal antibody and Nile-red stain were purchased from Sigma, St Louis, MO, USA. Apoptag®PLUS Peroxidase In Situ Detection Kit S701 was from Chemicon International, CA, USA. Rabbit polyclonal antibodies against human Bax, Bcl-2 and Bcl-x_L/S_ were from Santa Cruz Biotechnologies, Santa Cruz, CA, USA. Mouse anti-cyclin B1 monoclonal antibodies, mouse anti-cyclin E monoclonal antibodies, and rabbit anti-cyclin D1 monoclonal antibodies were from Cell Signaling Technology, Inc., Danvers, MA, USA. Annexin V-FITC was from Biosciences, USA. Chemiluminiscence system (ECL) was from Amersham Biosciences Argentina SS (Argentina). Nitrocellulose membranes were from Santa Cruz Biotechnologies, Santa Cruz, CA, USA. Multiwells were from TPP, Switzerland. All other reagents were of analytical grade.

## Methods

### Reverse transcriptase polymerase chain reaction (RT-PCR) analysis

Total cellular RNA was extracted using Trizol® according to the instructions of the manufacturer (GIBCO, Life Technologies, USA). Total RNA (2 μg) was added to the reverse transcription (RT) reaction mixture (20 μl) in the presence of oligo-dT primers and samples were incubated at 37°C for 60 minutes. The quality of each individual’s cDNA was confirmed by PCR with primers for β-actin producing bands of the expected size (data not shown).The primers used were employed previously by other workers for Kir6.1, Kir6.2, SUR1 [4], SUR2A and SUR2B [20]. PCR conditions were as follows: Kir6.1 forward (5′-CATCTTTACCATGTCCTTCC-3′) and reverse (5′-GTGAGCCTGAGCTGTTTTCA-3′), 336 bp; Kir6.2 forward (5′-GCTTTGTGTCCAAGAAAGG1-3′) and reverse (5′-CCAAAGCCAATAGTCACTTG-3′), 301 bp; 5 min 95°C, 35 cycles of 95°C for 20 s, 52°C for 45 s, and 72°C for 1 min. SUR1 forward (5′-CGATGCCATCATCACAGAAG-3′) and reverse (5′-CTGAGCAGCTTCTCTGGCTT-3′), 291 bp; SUR2A foward (5′-ATGCGGTTGTCACTGAAGG-3′) and reverse (5′-AATAGAAGAGACACGGTGAGC-3′), 215 bp; SUR2B forward (5′-GATGCGGTTGTCACTGAAGG-3′) and reverse (5′-TCATCACAATAACCAGGTCTGC-3′), 244 bp; 5 min 95°C, 35 cycles of 95°C for 1 min, 55°C for 1 min, and 72°C for 2 min. Reactions were terminated by final elongation step of 7 min at 72°C (Gene Amp PCR System 2400, PerkinElmer, MA, USA). Negative controls were performed with water instead of cDNA. PCR products were subjected to gel electrophoresis and detected by gel documentation system LumiBis DNR (Bio-Imaging Systems, Jerusalem, Israel).

### Cell culture

MBA-MB-231 cels were cultured in RPMI 1640 medium supplemented with 10% FBS, 0.3 g/l L-glutamine and 40 mg/l gentamicine and in the presence of Gli, Tam or vehicle. Cells were maintained at 37°C in a humidified atmosphere containing 5% CO_2_.

### Cell proliferation assay

For clonogenic assay, cells were seeded in 6-well plates (1.5×10^3^ cells/well) and incubated in the presence or absence of drugs for 10 days. Cells were treated with 10 to 50 μM Gli, 0.005 to 5 μM minoxidil; 0.1 to 5 μM tamoxifen; 0.01 to 0.1 nM doxorubicin or with the concentration of Gli that inhibited the proliferation to the 50% (IC_50_) plus different doses of minoxidil, tamoxifen or doxorubicin to assess the combined action of both drugs. Cells were then fixed with 10% formaldehyde in phosphate-buffered saline, PBS, stained with 1% toluidine blue in 70% ethanol. The clonogenic proliferation was evaluated by counting the colonies with 50 cells or more. The results are expressed as a percentage of control values.

### Determination of doubling time

For doubling time determination cells were seeded in 6-well plates (4×10^4^ cells/well), starved for 24 h and then incubated with IC_50_ Gli (25 μM) or vehicle for up to 96 h. Cells were trypsinized at 0, 1, 2, 3 and 4 days and counted using a hemocytometer. All experiments were performed in the logarithmic phase of cell growth. Triplicate plates were analyzed for each treatment and each time. The following formula was used to calculate the doubling time: N_t_ = N_0_ x e^kxt^, where N_0_ was the initial number of cells that increased exponentially with a rate constant, k. The doubling time (T) was calculated as: T = ln 2/k. The GraphPad Prism 5.0 software (GraphPad Software Inc., Philadelphia, U.S.A.) was employed.

### Cell cycle analysis by flow cytometry

Cells were cultured for 24 h without FBS. Synchronized cells were then treated with IC_50_ Gli (25 μM) or vehicle immediately after release from the block and harvested for up to 72 h. Then cells were collected by trypsinization, fixed with ice cold methanol, centrifuged and resuspended in 0.5 ml of propidium iodide (PI) staining solution (50 μg/ml PI in PBS containing 0.2 mg/ml of DNase-free RNase A). After incubation for 30 min at 37°C, samples were evaluated by flow cytometry (Becton Dickinson, USA). Cell cycle distribution was analyzed using Cylchred 1.0.2 software (Cardiff University, UK).

### Quantification of DNA synthesis

The quantification of cellular DNA synthesis was performed on cells by the addition of 30 μM BrdU for 2 h. Cells grown on sterile slides were washed with PBS and fixed with 10% formaldehyde in PBS. To denature the DNA into single-stranded molecules, cells were incubated with 3 N HCl for 30 min at room temperature. Then cells were washed in 1 ml of 0.1 M Na_2_B_4_O_7_, pH 8.5 to neutralize the acid and were then incubated overnight at 4°C with mouse anti-BrdU monoclonal antibody (1:50). Then, cells were incubated for 2 h at 37°C with FITC-conjugated anti-mouse IgG (1:100). After washed with PBS, cells were stained with DAPI (1:8000). Fluorescence was further visualized by fluorescence microscope. At least 1000 cells were scored for each determination.

### Apoptosis

Apoptotic MDA-MB-231 cells were detected after treatment with Gli or vehicle for 72 h. Phosphatidylserine exposure on the surface of apoptotic cells was detected by flow cytometry after staining with Annexin V-FITC and PI (50 μg/ml). Data were analyzed using WinMDI 2.8 software (Scripps Institute, CA, USA).

### Mitochondrial transmembrane potential

Variations of the mitochondrial transmembrane potential of the cells, ΔΨ_m_, were studied by means of the uptake of DiOC6, a specific fluorochrome that has been widely used in monitoring ΔΨ_m_. Cells were plated and treated 24 h after with 25 μM Gli for different incubation periods (24, 48 and 72 hs). The diluted dye at a final concentration of 40 nM in PBS was applied to cells for 15 min at 37°C. Cells were then washed twice with PBS, harvested and then analyzed by flow cytometry (Becton Dickinson, USA). Results were expressed as the percentage of mean fluorescence of respective controls.

### Determination of cell cycle and apoptosis related proteins

#### Flow cytometry

After treatment with Gli or vehicle for 72 h MDA-MB-231 cells were harvested, fixed with 4% formaldehyde and permeabilized with saponine. To evaluate intracellular protein content, cells were incubated with rabbit anti-Bcl-2 and rabbit anti-Bax antibodies. After washing, cells were incubated for 20 min with FITC-labeled anti-rabbit. The samples were analyzed with a FACScalibur flow cytometer (Becton Dickinson, USA). Data analysis was performed using WinMDI 2.8 software. For each sample 20,000 events were collected. The results are expressed as the percentage of respective control values.

#### Western blot assay

Cells were placed on ice and washed twice with cold PBS. Cells were then scraped into a lysis buffer (100 mM Tris/HCl buffer, pH 8, containing 1% Triton X-100 and protease inhibitors) and incubated for 15 min on ice. After centrifugation at 6000 rpm for 10 min, the supernatants were used for protein determination according to Bradford assay [21]. For Western blot, loading buffer (100 mM Tris/HCl buffer, pH 8, containing 1.7% sodium dodecyl sulfate (SDS), 0.02% bromophenol blue, 1.5% dithiotreitol, and 5% of glycerol) was added to samples and they were boiled for 3 minutes. Equal amounts of proteins (50 μg) were fractionated on SDS-polyacrylamide gels (12%) and transferred electrophoretically onto nitrocellulose membranes. Membranes were blocked and probed overnight with primary mouse anti-cyclin D_1_ (1:100), mouse anti-cyclin E (1:500), mouse anti-cyclin B (1:200), mouse anti-p27^Kip1^ (1:200), rabbit anti-Bax (1:500), rabbit anti-Bcl-x_L/S_ (1:500) and mouse anti-β-actin (1:1000) antibodies. Immunoreactivity was detected by using horseradish peroxidase-conjugated anti-mouse or anti-rabbit IgG, as appropriate, and visualized by enhanced chemiluminescence. Densitometric analyses were performed using the software Image J 1.32 J (NIH, USA).

### Evaluation of senescence-like phenotype

Senescence-associated β-galactosidase (SA-β-Gal) activity was detected in cells as previously described by Dimri [22] with some modifications. Cells treated with Gli (25 μM) or vehicle for 72 hs were fixed in 3.0% formaldehyde for 5 min, washed in phosphate buffered solution (PBS) and stained in 1 mg/ml X-gal solution at pH 6.0 for 4 hs at 37°C. SA-β-Gal positive cells were stained in blue. To visualize the cell architecture, the slides were counter-stained by haematoxylin and quantified by optical microscopy. At least 1000 cells were scored for each determination.

### Lipid accumulation

In order to examine the possible action of Gli in lipid accumulation, a classic terminal differentiation marker in mammary cells, the levels of neutral lipid were measured by flow cytometry using Nile-red staining [23]. Cells treated with Gli or vehicle for 2, 3 or 7 days were fixed and then incubated with Nile-red at a final concentration of 1 μg/ml in PBS for 20 min at room temperature. Cells were then analyzed by flow cytometry (Becton Dickinson, USA).

Data analysis was performed using WinMDI 2.8 software (Scripps Institute, CA, USA). The results were expressed as a percentage of control values.

### Cytostatic effect of glibenclamide

MDA-MB-231 cells (1x10^4^ cells/well) were plated into 6-well plates. After 24 h, cells were treated with 25 μM Gli or vehicle and detached with trypsin 3 or 7 days later. Cells were then counted and seeded in 6-well plates (1.2×10^3^ cells/well) in triplicates. After 10 days in culture, colonies were fixed with 10% buffered formalin and stained with 1% toluidine blue in 70% ethanol. The number of colonies was determined and normalized to the number of colonies in controls.

### Statistical analysis

In all cases the data shown are the means ± SEM of at least three independent experiments. Statistical analysis is indicated in each legend. Data were analyzed using the GraphPad Prism 5.0 (GraphPad Software Inc., Philadelphia, U.S.A.) and P values less than 0.05 were considered statistically significant.

## Results

### Expression of K_ATP_ channels in MDA-MB-231 cells

**Figure 1:**
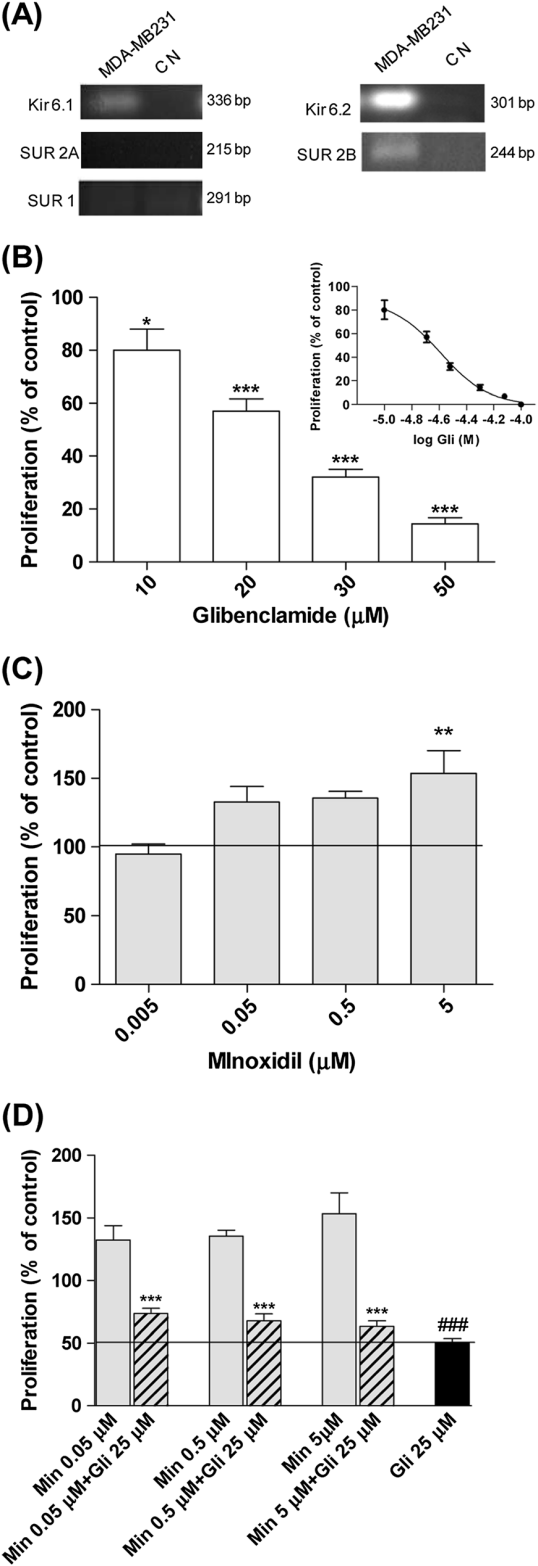
**Effect of Glibenclamide, a specific blocker of the K**_**ATP **_**channels, on cell growth. **The figure shows the mRNA expression of K_ATP _channels components and the effect of Gli and minoxidil (Min) on cell proliferation in MDA-MB-231 cells. Proliferation was evaluated by counting of colonies with 50 cells or more and expressed as percentage of values obtained with vehicle (means ± SEM of three experiments on parallel). Panel **A**: mRNA expression of K_ATP _channels in MDA-MB-231 cells by RT-PCR analysis. Agarose gel electrophoresis of PCR products showed bands corresponding to: Kir6.1 (336 bp), Kir6.2 (301 bp), SUR2B (312 bp). No bands were detected for SUR2A (215 bp) or SUR1 (291 bp). CN: negative control Panel **B**: Inhibition of proliferation obtained with different concentrations of Gli (10, 20, 30 or 50 μM). Insert shows the dose–response curve used to determine IC_50 _(IC_50_ = 25 μM). Panel **C**: Increase of proliferation obtained with Min 0.05; 0.5 or 5 μM. Panel **D**: Results obtained with IC_50 _Gli plus different concentration of Min. Panel **B **and **C**: *p < 0.05 vs. control; **p < 0.01 vs control; ***p < 0.001 vs. control, One way ANOVA and Dunnet post test. Panel **D**: ***p < 0.01 vs. Min 0.05 μM; vs. Min 0.5 μM; vs. Min 5 μM. ^###^p < 0.001 vs. Min 0.05 μM; vs. Min 0.5 μM; vs. Min 5 μM. One way ANOVA and Tuckey post test.

Though many reports describe the presence of different potassium channels in diverse human cancer cell lines, at present there is little evidence about the expression of K_ATP_ channels in breast cancer cells. Since Gli is a specific blocker of K_ATP_ channels, the expression of mRNA for different subunits (Kir6.1, Kir6.2 and SURs) was examined by RT-PCR in MDA-MB-231 cells. As shown in Figure 1A bands of 336 and 301 bp for Kir6.1 and Kir6.2 respectively were detected after electrophoresis, indicating gene expression of pore components for at least two channel types in this cell line. Furthermore, a predicted band of 244 bp was found indicating the expression of SUR2B gene. The expression of SUR1 and SUR2A genes was not detected (291 bp and 215 bp, respectively). Altogether these results indicate that pore-forming and regulatory subunits are expressed in this cell line. ERα (+) MCF-7 breast cancer cells were also analyzed and Kir6.1, Kir6.2 and SUR1 were found expressed in this cell line (data not shown).

### Effect of glibenclamide on cell proliferation

The effect of Gli on cell proliferation was tested by means of a clonogenic assay. A significant concentration dependent inhibition on cell growth was observed when Gli was added to cell cultures in concentrations over 10 μM; the IC_50_ value was 25.6 ± 3.2 μM (Figure 1B). The increased doubling time (T value, Table 1) obtained in the presence of 25 μM Gli is in concordance with the inhibition of proliferation previously demonstrated using the clonogenic assay.

**Table 1:** Determination of cell doubling time

**Treatment**	**Duplication Time (hs)**
**Control**	**24.0 ± 4.3**
**Gli**	34.6 ± 4.5 *

Gli: 25 μM gliblenclamide. * p < 0.05 vs control, *t* test.

In order to support the hypothesis of K_ATP_ channels involvement in MDA-MB-231 cell proliferation we used minoxidil, a well known specific opener of these channels. The results showed an increase in cell clonogenic growth for concentrations over 0.05 μM, which became significant at 5 μM (Figure 1C). Figure 1D shows that the increment in proliferation produced by the channel opener was totally reversed by 25 μM Gli.

**Figure 2:**
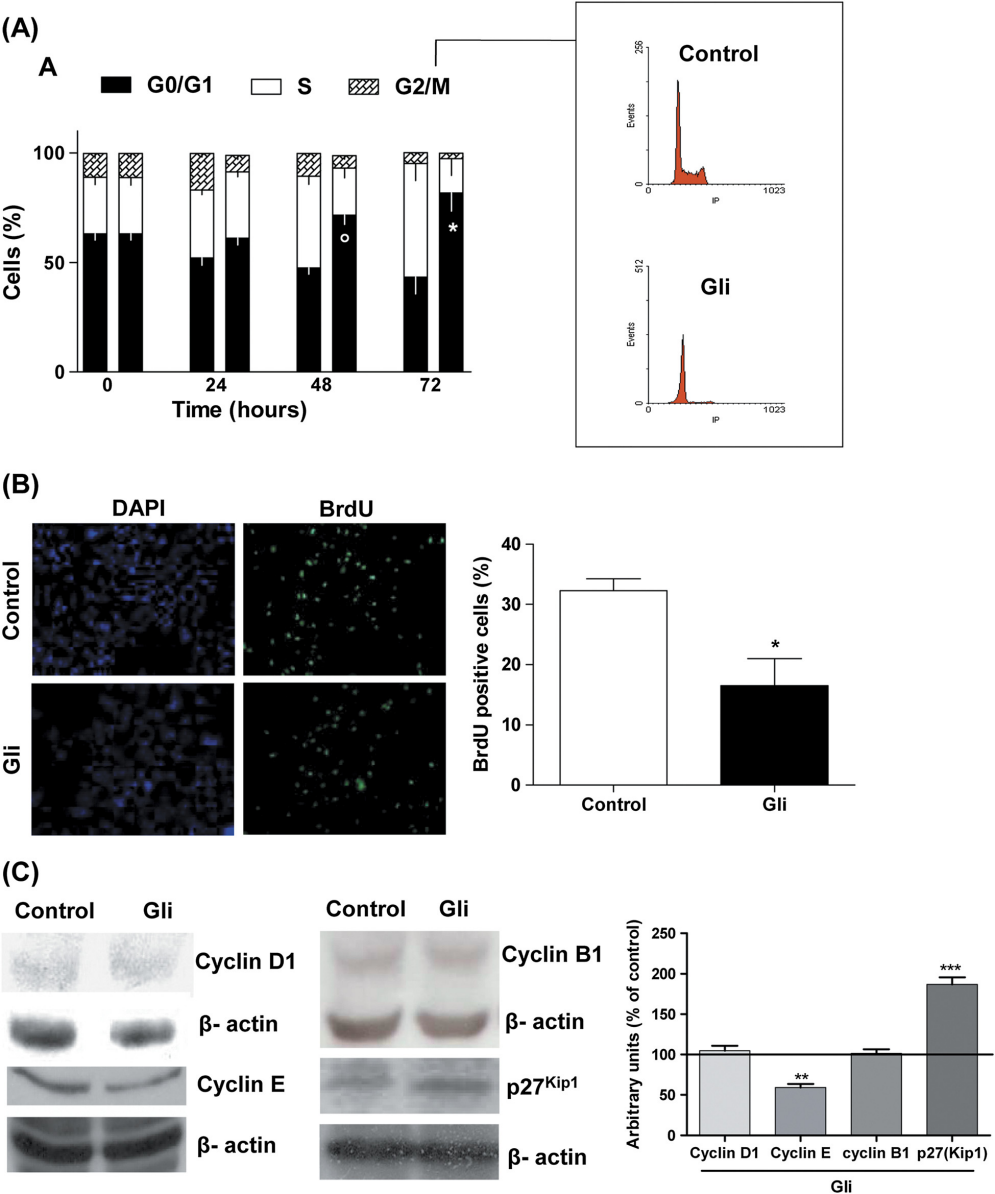
**Effect of Glibenclamide on cell cycle progression. **Panel **A**: Synchronized MDA-MB-231 cells were treated with IC_50 _Gli (25 μM) or vehicle for 24, 48 or 72 h and the fraction of cells in each phase of cell cycle was evaluated by flow citometry. Gli treatment clearly arrested cells at G0/G1 phase. Results are expressed as percentage of the value obtained with vehicle (means ± SEM of three experiments on parallel). °p < 0.01 vs control; *p < 0.001 vs control, *t* test. Left bars: control; right bars: Gli-treated cells. Panel **B**: A decrease in BrdU incorporation to DNA was observed when cells were treated with 25 μM Gli for 48 h. Results are expressed as the means ± SEM of three experiments on parallel. *p < 0.05 vs. control, *t *test. Panel **C**: Expression of G1-S regulatory proteins in MDA-MB-231 cells treated with Gli or vehicle for 72 h was analyzed by Western blot. Gli decreased the level of cyclin E and increased p27^Kip1^. Representative immunoblot images of cyclins D1, B1, E and p27^Kip1 ^are illustrated. Relative quantification was performed by densitometric analyses. Actin densitometric values were used to standardize for protein loading. Bars represent the mean ± SEM of three independent experiments. **p < 0.01 vs control; ***p < 0.001 vs control, *t* test.

The analysis of cell cycle phase distribution demonstrated that Gli produces a significant increase in the number of cells in G1 phase at 24, 48 and 72 h post treatment, clearly demonstrating a significant G0/G1 cell cycle arrest (Figure 2A). A consequent decrease in cells in S and G2 phase versus control was also observed. Consistent with these observations, Gli inhibited the active DNA synthesis when it was evaluated by BrdU incorporation (Figure 2B).

The expression of proteins implicated in the control of different phases of the cell cycle was investigated by Western blot analysis. Studies of proteins specifically related with phase G1 of cell cycle demonstrated that 25 μM Gli reduced expression of cyclin E whereas cyclin D1 remained unchanged after 72 h of treatment. Furthermore, p27^Kip1^ levels were up-regulated in the same experimental conditions. In addition, the level of cyclin B1 expression, which is involved in the control of G2-M transition, was not modified by Gli treatment.

### Effect of glibenclamide on cell death

**Figure 3:**
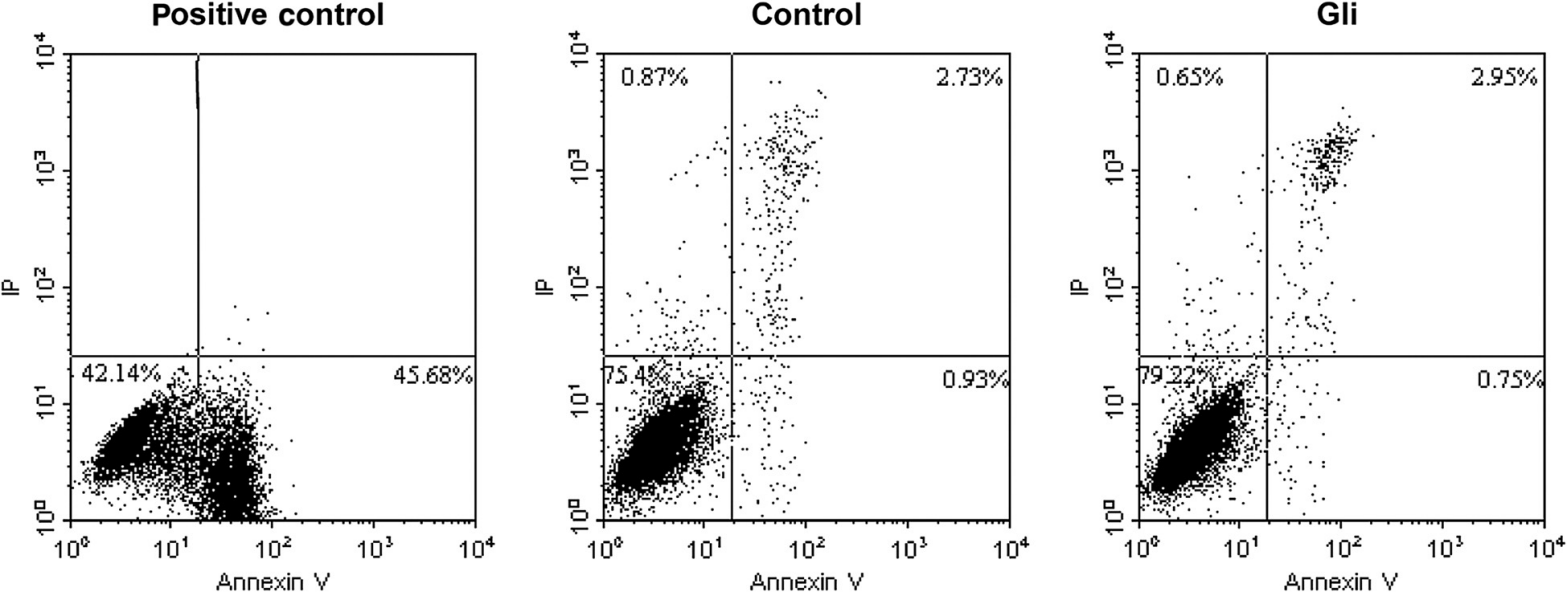
**Evaluation of apoptosis by Annexin-V method. **Apoptosis was assessed after incubating the cells with Gli or vehicle by 72 h. For positive control cells were treated with H_2_O_2 _(5 mM) for 30 minutes. Fluorescence was evaluated immediately after Annexin-V staining by flow cytometry. Gli (25 μM) did not induce an increment in apoptosis of MDA-MB-231 cells. Positive Annexin-V cells are shown in both right quadrants.

To determine if the decrease in proliferation exerted by Gli could be due to an apoptotic effect, we assessed apoptosis by two different methodologies. Results showed that Gli did not increase the number of apoptotic cells by Annexin-V staining (3.66 ± 0.62% in control vs 3.70 ± 0.69%) after 72 h of treatment (Figure 3). In accordance, neither it produced the disruption of the mitochondrial transmembrane potential (ΔΨ_m_) that is associated with mitochondrial dysfunction and linked to cell death and loss of cell viability (Table 2).

**Table 2:** **Effect of Gli on mitochondrial transmembrane potential (ΔΨ**_**m**_**)**

**Time (hs)**	**ΔΨm (% of control)**
**24**	**99.7 ± 7.3**
**48**	**97.0 ± 3.9**
**72**	**94.3 ± 12.7**

Gli: 25 μM glibenclamide. p:NS vs. control, One way ANOVA.

**Figure 4:**
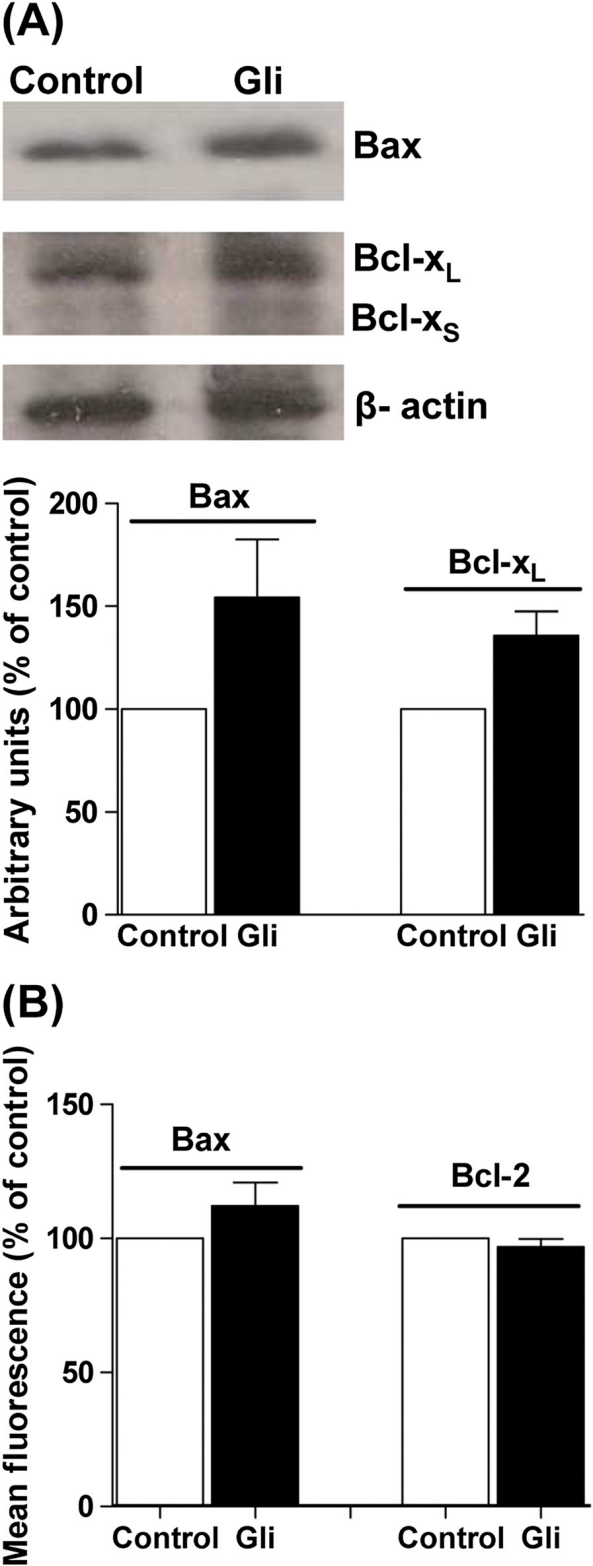
**Expression of proteins involved in apoptosis. **Panel **A**: Bax and Bcl-x_L/S _expression determined by Western Blot employing specific antibodies in MDA-MB-231 cells treated for 72 h with 25 μM Gli (Gli) or vehicle (control) cells. The figure shows a representative Western blot of three independent experiments and the quantification of bands obtained for Bax and Bcl-x_L/S _protein. Bars represent the mean ± SEM of three independent experiments. p: Non significant (NS), *t *test. Panel **B**: Expression of Bcl-2 and Bax protein in MDA-MB-231 cells treated by 72 h with 25 μM Gli (Gli) or vehicle, obtained by flow cytometry. Bars represent the mean fluorescence ± SEM obtained by three independent experiments. p: NS, *t *test.

It is known that the Bcl-2 family of mitochondrial proteins is strongly linked to the process of apoptotic cell death; some members of the family act as antiapoptotic proteins such as Bcl-2, Bcl-x_L_, while others act as inductors of cell death as Bcl-x_S_ and Bax [24-26]. We determined the expression of these proteins by flow cytometry and Western blot. By both methodologies we showed that 72 h after treatment with 25 μM Gli, the level of expression of pro-apoptotic protein Bax was slightly increased in relation to control at the same time, although this increase was not statistically significant (Figure 4A and 4B). On the other hand, antiapoptotic Bcl-2 protein did not modify its expression when cells were treated with Gli (Figure 4B). The pro-apoptotic isoform, Bcl-x_S_, showed a very low expression while the antiapoptotic isoform Bcl-x_L_ expression levels were higher but did not significantly change with Gli-treatment (Figure 4A).

**Figure 5:**
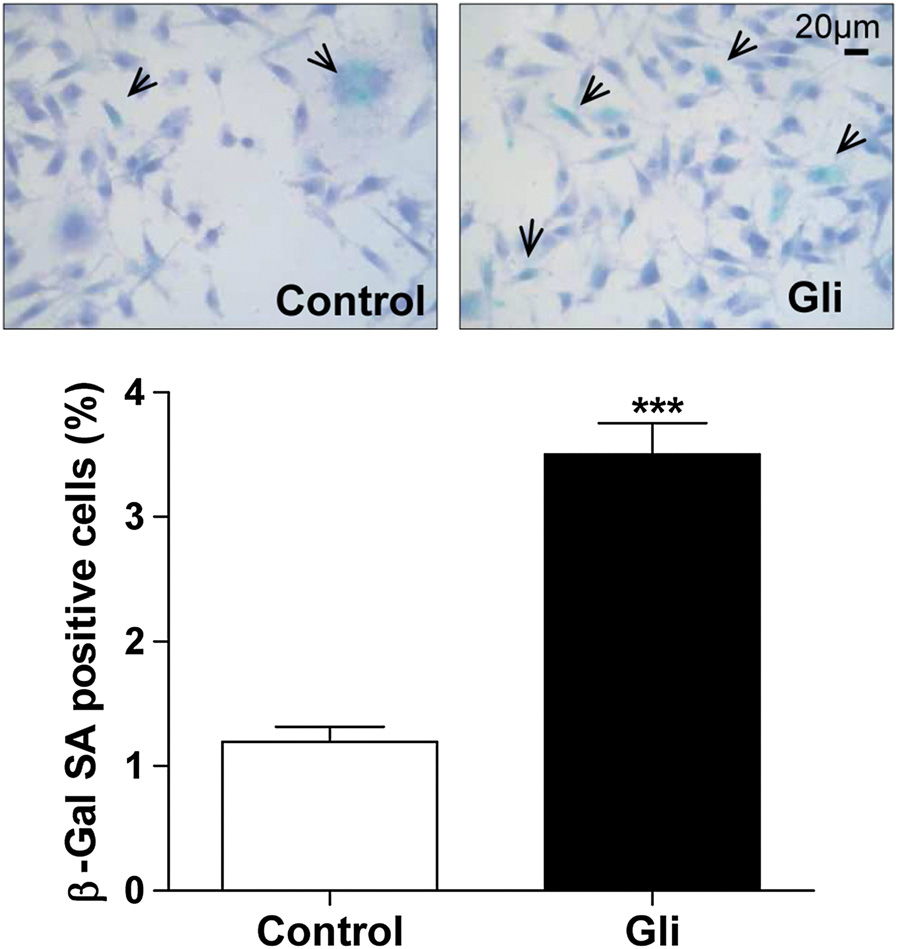
**Evaluation of Senescence. **MDA-MB-231 cells were cultured for 48 h with 25 μM Gli or with vehicle. Senescence was assessed by the activity of SA-β-GAL. Panel **A**: representative photographs where positive SA-β-GAL cells are indicated by arrows. Gli produces an increment in cell senescence. Panel **B**: Percentage of cells SA-β-GAL positive were calculated by counting of at least 1000 cells (630X). Bars represent the mean ± SEM of three independent experiments. *p < 0.001 vs. control, *t* test.

We also evaluated the induction of cell senescence as a mechanism of cell death. To identify the senescent cells, senescence-associated β-galactosidase (SA-β-GAL) activity was assessed. MDA-MB-231 cells treated with 25 μM Gli showed an increase in the percentage of senescent cells versus those treated with vehicle (3.5 ± 0.4% vs 1.2 ± 0.2%; Figure 5).

### Effect of glibenclamide on cell differentiation

Differentiation is one possible mechanism involved in the loss of cell proliferative ability. In mammary cells the accumulation of neutral lipids in cytoplasm is a specific marker of this process. We evaluated by flow cytometry the content of neutral lipids using Nile-red staining and results demonstrated no differences between control and 25 μM Gli treated cells up to 7 days (Table 3). Sodium butyrate, an effective differentiation agent in ERα (+) and ERα (−) breast cancer cells [27], was used as positive control.

**Table 3:** Effect of Gli on neutral lipid accumulation

**Time (days)**	**Mean Fluorescence of Nile Red (% of control)**	
	**Gli**	**Na Butyrate**
**2**	**92.3 ± 4.7**	134.3 ± 6.7 ***
**3**	**99.3 ± 1.7**	165.7 ± 7.3 ***
**7**	**99.0 ± 10.1**	192.7 ± 5.3 ***

Gli: 25 μM glibenclamide. Na Butyrate: 10 mM Sodium butyrate as the positive control. p: NS Gli vs control; *** p < 0.001 butyrate vs control, One way ANOVA and Dunnet test.

### Cytostatic effect of glibenclamide

**Figure 6:**
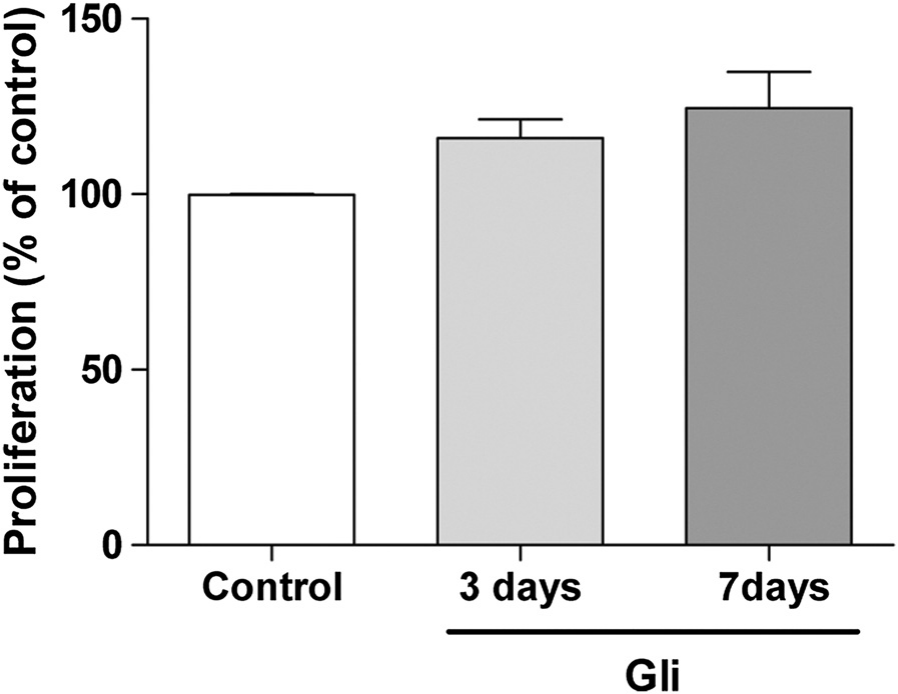
**Evaluation of cytotoxic or cytostatic effect. **MDA-MB-231 cells treated with 25 μM Gli or vehicle for 3 or 7 days were re-seeded at low density to evaluate clonogenic capacity. After 10 days in culture, the number of colonies was determined and normalized to the number of colonies in controls. Bars show that Gli pre-treatment did not signifcantly affect clonogenic capacity. p: NS, One way ANOVA.

To determine whether the growth inhibitory effect was reversible, a characteristic of cytostatic agents, cells were treated with 25 μM Gli or vehicle for 3 or 7 days and then they were trypsinized and re-plated at low density in the absence of any treatment to assess clonogenic proliferation. Results in Figure 6 showed that there is no significant difference between Gli-pretreated cells for different time periods and control cells, suggesting that the antiproliferative effect of Gli is elicited only when the drug is present and it does not involve cell toxicity.

### Combination treatments

**Figure 7:**
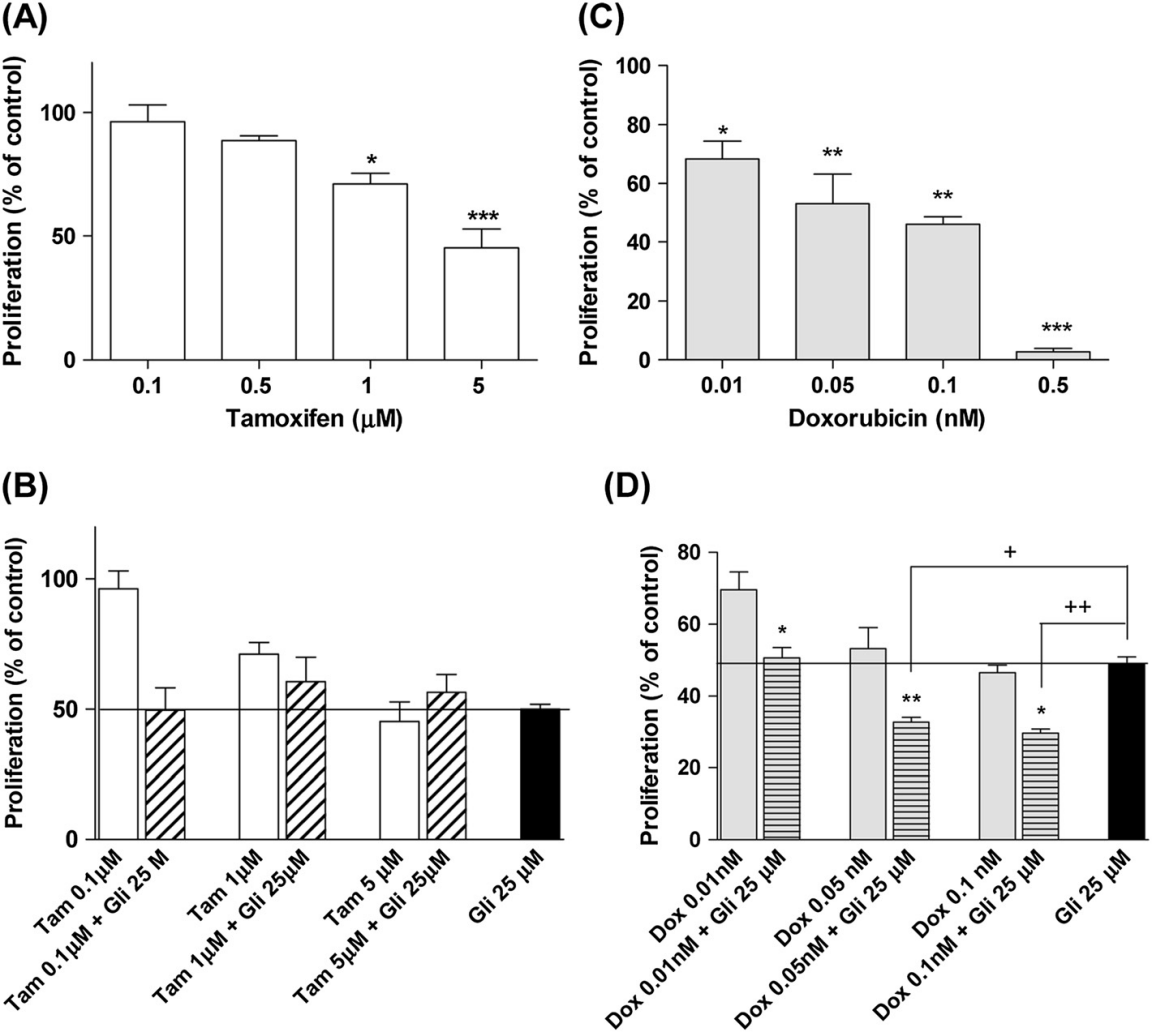
**Glibenclamide combination with antineoplasic drugs. **MDA-MB-231 cell proliferation was evaluated by counting of colonies with 50 cells or more and expressed as percentage of controls (means ± SEM of three independent experiments). Panel **A**: Results obtained with tamoxifen (Tam) 0.1, 0.5, 1 or 5 μM. Panel **C**: Inhibition of proliferation obtained with doxorubicin (Dox) 0.01, 0.05, 0.1 or 0.5 nM. Panel **B **and **D**: Results obtained for combination of 25 μM Gli plus different concentrations of Tam or Dox. Panel **A **and **C**: *p < 0.05 vs. control; **p < 0.01 vs. control; ***p < 0.001 vs. control, ANOVA and Dunnet post test. Panel **B**: p: NS vs. Gli 25 μM, *t* test. Panel **D**: *p < 0.05 vs. Dox 0.01 nM, vs. Dox 0.1 nM; **p < 0.001 vs Dox 0.05 nM; ^+^p < 0.05 vs. Dox 0.05 nM + Gli 25 μM; ^++^p < 0.001 vs. Dox 0.1 nM + Gli 25 μM. One way ANOVA and Tuckey post test.

We assayed the combination of Gli with tamoxifen or doxorubicin to explore a possible increase in efficacy. The growth of MDA-MB-231 cells was inhibited by tamoxifen with an IC_50_ equal to 5 μM (Figure 7A). The concentration of Gli that inhibited cell growth in a 50% (25 μM) was used to evaluate the combined action of Gli plus tamoxifen. The inhibitory effect exerted by the combination of Gli plus tamoxifen was similar to that observed for Gli alone (Figure 7B).

Doxorubicin also inhibited cell proliferation in a concentration dependent manner (Figure 7C). The combination of 25 μM Gli plus doxorubicin in doses over 0.05 nM was more effective to inhibit cell proliferation than single treatments (Figure 7D).

## Discussion

Different subtypes of potassium channels have been shown to be directly implicated in normal and malignant cell proliferation [9]. Some of these channels are overexpressed in tumors and therefore they are potential targets for anticancer therapies [28].

We have previously demonstrated that Gli exerts an antitumoral action on NMU-induced mammary tumors in rats, which is an experimental model similar in ER expression and hormone-dependence to human breast cancer [29,30]. In these tumors Gli action was potentiated by the combination with tamoxifen [31]. In this paper we analyzed the effect of Gli in MDA-MB-231 ERα (−) breast cancer cell proliferation.

We studied the expression of mRNA for the different subunits that constitute K_ATP_ channels in MDA-MB-231 cells and determined that these cells express both pore forming subunits, Kir6.1 and Kir6.2, and the regulatory subunit SUR2B. Coincidently, Bondestine et al. have recently reported the expression of SUR2 protein in MDA-MB-231cells, while SUR1 could not be detected in this cell line [32]. In consequence two whole octameric functional channels could be present in MDA-MB-231 cells. In specific tissues different subunit combinations have been detected, e.g., pancreatic β cells and neurons have Kir6.2/SUR1 channels, whereas skeletal muscle expresses Kir6.2/SUR2A channels. These differences imply that drugs may have distinct abilities to affect K_ATP_ channels in diverse tissues depending on the type of SUR expressed [20]. Gli binds to every type of SUR and therefore inhibits the activity of all known K_ATP_ channels [33]. When we assayed the effect of Gli on MDA-MB-231 cell proliferation, results showed that Gli inhibits clonogenic ability in a concentration-dependent way, with an increment in population doubling time. It has been reported that the potassium-dependent changes in membrane potential play a crucial role in the proliferation of many types of normal and tumor cells. The opening of potassium channels in the cell membrane produces a hyperpolarization of membrane potential which is required for the progression through the cell cycle. As a consequence in the presence of potassium channel blockers cell proliferation is inhibited [34,35]. Woodfort et al. early determined that different potassium channel antagonists, as Gli, produce a concentration-dependent growth inhibition on MCF-7 cells with a significant arrest of cells in G0/G1 phase [36]. Diverse drugs signaled as specific K_ATP_ channel openers which include minoxidil, pinacidil and diaxozide augment DNA synthesis and proliferation of normal and tumor cells [37-39]. In the experiments using minoxidil we demonstrated an increase in MDA-MB-231 cell proliferation in concentrations over 0.05 μM. In addition when different concentrations of minoxidil were combined with 25 μM Gli the increment in proliferation was totally reverted. Taken together, these results suggest that Gli could reduce cell proliferation in MDA-MB-231 cells acting through K_ATP_ channels.

The analysis of cell cycle progression indicated that 25 μM Gli produces an arrest in the G0/G1 phase of cell cycle after 48 h of treatment. After release of serum starvation, the diminution of the proportion of cells in S phase in Gli treated cultures was confirmed by the decrease in BrdU incorporation. The cell division cycle integrates several processes and signal transduction pathways to commit the progression of a cell through or its arrest in a specific cell cycle phase. It is generally accepted that the cell cycle regulators cyclin D1 and cyclin E play an important role in early G1 and late G1 progression. In addition, the progression is tightly regulated by the respective cyclin activated subunits cyclin-dependent kinases (Cdks) and their inhibitors (Cdkis). p27 is a member of the Kip/CIP family of Cdkis known to act in the G1 phase of the cell cycle, preventing G1-S transition [40,41]. The study of proteins involved in the progression through the phases of cell cycle by immunoblot, has evidenced a decrease in cyclin E expression with a raise in cyclin inhibitor p27^Kip1^ in Gli treated MDA-MB-231 cells. It is known that the activity of cyclin E in conjunction with its kinase subunit Cdk2, is limiting for the passage of cells through the restriction point needed for the progression of cells from G1 into S-phase [42]. p27^Kip1^ binds to the cyclinE/Cdk2 complex and inhibits the kinase thus impeding G1-S passage [43]. Altogether our results suggest that Gli inhibits MDA-MB-231 cell proliferation by hindering G1-S transition. Other authors have also reported that different potassium channels blockers and other agents that cause cell membrane depolarization, produce the arrest of cells in G1 phase with the involvement of different cyclins and inhibitors depending on the cell type [34]. Eto reported that various anti-cancer agents specifically up-regulate p27^Kip1^ expression without affecting expression of the other regulatory proteins of G1-S cell cycle transition in human breast cancer cell lines. Moreover, in concordance with our work, he reported that the up-regulation of p27^Kip1^ expression in these cell lines by anti-cancer agents linearly and positively correlates with the degree of growth inhibition of NMU-induced mammary tumors by the same anti-cancer agent [44].

It has been suggested that chemotherapeutic agents can prevent mammary carcinogenesis and tumor growth through different mechanisms that include apoptosis, differentiation and senescence. Regardless of the mechanism, the evaluation of new antitumoral drugs has as a goal to stop the cell proliferation and produce the death of the tumor cell by apoptotic or non-apoptotic means [45]. Previously, working with NMU-induced mammary tumors in rats, we reported that Gli clearly produced the inhibition of tumor growth through a decrease in cell proliferation and an increase in cell apoptosis and differentiation [31]. It has been reported that Gli produces apoptosis in malignant cell lines such as hepatoblastoma and gastric cancer cells [6,7]. Furthermore, Iwakura and coworkers found that Gli produces a sustained increase in the entrance of Ca^2+^ to the cells inducing their death through apoptosis [46]. On the contrary, we demonstrated that Gli neither produced apoptosis nor triggered the early events of this mechanism of cell death in MDA-MB-231 cells. We also studied the expression of apoptosis related proteins and determined that Gli did not significantly affect the ratio of the expression of Bax/Bcl-2 proteins at any time evaluated. The relation between antiapoptotic and apoptotic proteins of Bcl-2 family is a better determinant of the susceptibility to apoptosis than the expression of each member separately.

In view that some chemotherapeutic agents are able to produce cell senescence as part of their mechanism of action [45], this possible way of action was studied in the MDA-MB-231 cells treated with Gli. Data obtained in our experiments indicate that there is a slight increase in cell senescence after Gli treatment. However, Gli did not result cytotoxic for neoplastic MDA-MB-231 cells so they clearly keep their clonogenic capacity after being exposed to Gli for seven days. Our results are in agreement with the reported by Woodfork et al., showing that Gli induced a cell cycle arrest in G0/G1 phase in MCF-7 cells that could be reverted by the removal of the drug [36].

Mammary tumor cell differentiation is characterized by an arrest in cell proliferation, nuclear and cytoplasmatic morphological changes and also by the increased expression of the components of milk such as lipids [47,48]. The accumulation of neutral lipid drops in the cell cytoplasm is used as a marker of this process. In our research Gli did not induce differentiation in MDA-MB-231 cells after treatment for seven days.

Antiestrogens such as tamoxifen are widely used for the treatment of ERα (+) breast cancer. Nevertheless, tamoxifen may elicit pro-apoptotic effects in ERα (−) breast cancer cells by the modulation of various cell signaling pathways in an ER-independent manner [49-53]. However, these effects have generally been reported when relatively high concentrations of tamoxifen were used. In the present work we used tamoxifen in combination with Gli as a strategy to enhance its action in ERα (−) breast cancer even if Gli acts as cytostatic in MDA-MB-231 cells. In our experiments tamoxifen inhibited proliferation significantly at quite high concentrations (5 μM) as expected and the combination treatment did not produce a higher inhibitory effect on cell proliferation than each treatment alone. Abdul et al. demonstrated that two nonspecific potassium channels blockers, amiodarone and dequalinium, potentiated the growth inhibitory effects of tamoxifen on human breast, prostate and colon cancer cell lines [38]. In view of our previous results in NMU-induced mammary tumors [31] it could be suggested that Gli may potentiate tamoxifen action only in breast hormone-dependent cells.

Doxorubicin is extensively used in chemotherapy for patients with metastatic breast cancer. In spite of its excellent anti-tumor activity, the associated acute and chronic toxicities lead to a relatively low therapeutic index [54]. Therefore, combination treatment with a non-toxic drug which can lower the dose would be advantageous. Our results indicate that the combined treatment of Gli and doxorubicin displayed an additive anti-proliferative effect. In this regard, Gli was found to inhibit multidrug resistance protein (MRP1) activity in human lung cancer cells [55]. In addition it has been also demonstrated that the overexpression of the inwardly rectifying K channel Kir2.2 decreased doxorubicin-induced reactive oxygen species accumulation and cell growth inhibition in several cancer cell lines [56]. Further studies are to needed to fully investigate the mechanism involved in the antiproliferative response of MDA-MB-231 cells to the combined treatment glibenclamide with doxorubicin.

## Conclusions

Gli, a drug widely used in clinics for the treatment of type 2-diabetes, is atoxic at doses routinely employed and of low cost. Our experimental data clearly demonstrated that it produces a cytostatic effect in MDA-MB-231 cells inhibiting the G1-S phase progression without inducing cell death or differentiation. Nevertheless, in spite of the lack of cytotoxic action, the interesting observation about the effect of the combination of Gli with doxorubicin on proliferation warrants an exhaustive research to elucidate the pathways involved in this interaction, leading to the consideration of a novel role for Gli as an adjuvant in breast cancer treatment.

## Competing interests

The authors declare that they have no competing interests.

## Authors’ contributions

MN is Ph.D and she designed the study, performed the experiments, interpreted the data, and wrote the manuscript. VM, GC, and CC are Ph.D. and carried out clonogenic and flow cytometry assays and participated in data analyses and interpretation. RB and GM are Ph.D. and participated in data analysis and discussion and critically revised the manuscript. MC carried out microscopic observation. ER performed the statistical analysis. All authors have expertise on radiopharmacology and receptors study. VM, CC, GM and RB are members of the National Research Council (CONICET). MN, VM, GC, CC, ER, RB, and GM are professors in the University of Buenos Aires. MC is M.D. and he is specialist in anatomopathology. All authors read and approved the final manuscript.

## Pre-publication history

The pre-publication history for this paper can be accessed here:

http://www.biomedcentral.com/2050-6511/14/6/prepub
